# Life-safety disparity index: a comprehensive, valid, and reliable tool to assess Chinese people’s life-safety performance

**DOI:** 10.3389/fpubh.2025.1476051

**Published:** 2025-06-04

**Authors:** Xiaoyue Ge, De an Wu, Xinming Shi, Junxiang Zhang, Jinghui Jiang, Bicheng Zhang, Hongguang Wang

**Affiliations:** ^1^The Academy of Chinese Health Risks of West China Hospital, Sichuan University, Chengdu, China; ^2^School of Mathematics, University of Electronic Science and Technology of China, Chengdu, China

**Keywords:** life-safety, health, life-safety disparity index, model, China, life-safety guarantee zone

## Abstract

**Aims:**

In order to accurately and quantitatively grasp the current situation and trend of Chinese people’s life-safety performance and to provide scientific basis and suggestions for the government, scientific research institutions, enterprises and the public to do a good job in life-safety protection.

**Methods:**

The establishment process of life-safety disparity index (LDI) included six steps, including (i) concept building (ii) framework formulation; (iii) indicator selection; (iv) database building; (v) weight determination; and (vi) LDI scores calculation. The indicator scheme for LDI based on this framework was constructed after multiple rounds of panel discussions with our expert advisory committee. We adopted the Data envelopment analysis-Compromise planning (DEA-CP) model to determine the weights for each indicator and calculate score.

**Results:**

The weighted indicator scheme of LDI comprised 6 first-grade indicators, 21 s-grade indicators, and 65 third-grade indicators. According to the pilot analysis based on the data from 31 provinces of China mainland, the maximum value of LDI was 100.00, the minimum value was 54.75, and the maximum value was 1.83 times the minimum value. According to the score, China mainland was divided into 5 life-safety guarantee zones, including high security zone, medium to high security zone, medium security zone, low to medium security zone, and low security zone.

**Conclusion:**

LDI, which has been strictly verified, would represent the world’s first assessment tool that builds a conceptual framework from the overall perspective of ensuring life-safety.

## 1 Introduction

In 2000–2019, before the COVID-19 pandemic in the new millennium, the world has achieved remarkable progress in health. The child mortality has reduced by half, the maternal mortality rate has dropped by one third, the incidence rates of many infectious diseases have declined, and the risk of premature death caused by noncommunicable diseases and injuries has declined. The global life expectancy at birth has risen from 67 years in 2000 to 73 years in 2019 (1).

The COVID-19 has had an unprecedented impact on every country in the world. Up to 28 of April 2024, a total of 775.38 million people worldwide have been infected and 7.05 million people have died (2). According to WHO data, years of life lost estimates show that a total of 336.8 million life-years have been lost globally due to the COVID-19 pandemic in 2020–2021. The global life expectancy at birth has dropped from 73 years in 2019 to 71.3 years in 2021 (3). The outbreak of COVID-19 has made countries all over the world pay more attention to ensuring people’s life-safety and biosecurity.

To achieve the goal of universal coverage of health care in China, the new round of health sector reform announced in 2009, a series of strategies and measures were proposed, summarized as “four beams and eight pillars”(*Si Liang Ba Zhu*) (4). As of 2011, the health insurance scheme reached a high point in coverage of 95%. The average life expectancy of the Chinese has increased from 35 years in 1949 to 78.6 years in 2023 (5). There is a large gap in the life expectancy of different provinces in China. Shanghai has the longest life expectancy, which is 82.55 years, while Tibet has the shortest life expectancy, which is 72.19 years, with a difference of 10.36 years. The serious inequality of this indicator shows that there are still many problems in ensuring the life-safety of people in China. As a developing country with a population of 1.408 billion, accounting for 17.4% of the global population, ensuring the safety of every individual in China poses a significant challenge. Recognizing this challenge, we propose six steps toward development of the Life-safety Disparity Index (LDI) as a potential tool for the systematic evaluation of life-safety performance in various provinces of China.

## 2 Methods

LDI was constructed in six steps, including (i) concept building (ii) framework formulation; (iii) indicator selection; (iv) database building; (v) weight determination; and (vi) LDI scores calculation.

### 2.1 Concept

According to the WHO’s latest charter, health is a state of physical, mental and social perfection, not just the absence of disease or frailty (6). Health care and medicine are important means to protect human health.

Life-safety usually refers to the state and ability of human beings to deal with themselves, natural, human and other adverse factors, and to ensure their normal life and social activities. The self-factors include lifestyle, diet, aging, metabolism, etc. The natural factors include climate, hydrology, geology, altitude, etc. The human factors include biological laboratory leakage, biological terror, biological warfare, etc. Health is the inherent requirement and the core expression of life-safety.

Biosecurity refers to the state and ability of a country or region to effectively respond to the impacts and threats caused by all dangerous organisms, as well as the abuse and misuse of biotechnology, in order to maintain and safeguard people’s life-safety, ecological security and national security. Biosecurity includes public health security, which core is to protect people’s life-safety, agricultural biosecurity, biodiversity and ecological security, biotechnology and laboratory security, national border biosecurity, national defense biosecurity, biosecurity supportability building (7).

Most institutions and experts generally endorse that national security refers to a stable state of no external aggression and threats, no internal chaos and hidden dangers (8). Biosecurity is a part of China’s national security.

LDI refers to the gap in the ability and effect of ensuring life-safety between different countries, regions and departments, mainly including the quantity, quality, structure and benefit of health level, medical capacity, disease prevention and control, ecological environment, medical expenditure, health industry, public health policies, etc.

### 2.2 Index framework formulation

We developed the framework based on the concept of life-safety and LDI, including 6 core components: health level, medical capabilities, disease prevention and control, ecological environment, health expenditure and health industry.

### 2.3 Indicator selection

We constructed 73 initial indicators after four rounds of expert consultation. In the first round, based on the framework, the first, second, and third grade indicators were preliminarily determined. In the second round, large-scale collection of indicator data begined based on core data sources. In the third round, we integrated indicators based on the collected indicator data and condense the number of indicators. In the fourth round, we further optimized the indicators using proportion algorithms. However, due to high data missing or lack of official data, 8 indicators were deleted, ultimately forming an indicator system with 6 first-grade indicators, 21 s-grade indicators, and 65 third-grade indicators.

### 2.4 Data sources

The raw data used in this study, except for the altitude from the topographic map (topographic map), were all from the China Health Statistics Yearbook, China Statistical Yearbook, Urban and Rural Construction, Statistical Analysis Report on the Operation of the Drug Distribution Industry released by the Ministry of Commerce, the Ecological Environment Statistical Bulletin and other report released by government and the relevant departments.

### 2.5 Weight determination and LDI scores calculation

LDI used an improved Data envelope analysis-Compromise planning (DEA-CP) model (9). The advantage of this model was that it could intelligently determine the weight of each indicator to avoid the subjective error of artificial weight determination. The score of the best performing life-safety unit (country, region, department, institution, etc.) in the same indicator was 100. Afterwards, comparing the remaining units with the optimal unit and calculating the gap between different units for the same indicator. The LDI was a comprehensive calculation of the gap between multiple indicators. It is a two-stage comprehensive evaluation model:

First Stage: The traditional DEA model is used to calculate the optimal efficiency score for each life-safety unit (country, region, department, institution, etc.), allowing each unit to adopt the weights most favorable to itself, resulting in an ideal score vector.
Second Stage: Compromise Programming is employed to minimize the overall deviation between the actual scores of all evaluation units and the ideal scores. The L2 norm (Euclidean distance) is used for optimization, solving for a unified weight within the feasible weight domain to ensure fairness and consistency, avoiding the issue of weight dispersion in DEA.

## 3 Results

### 3.1 LDI indicator and weight scheme

**Figure 1:**
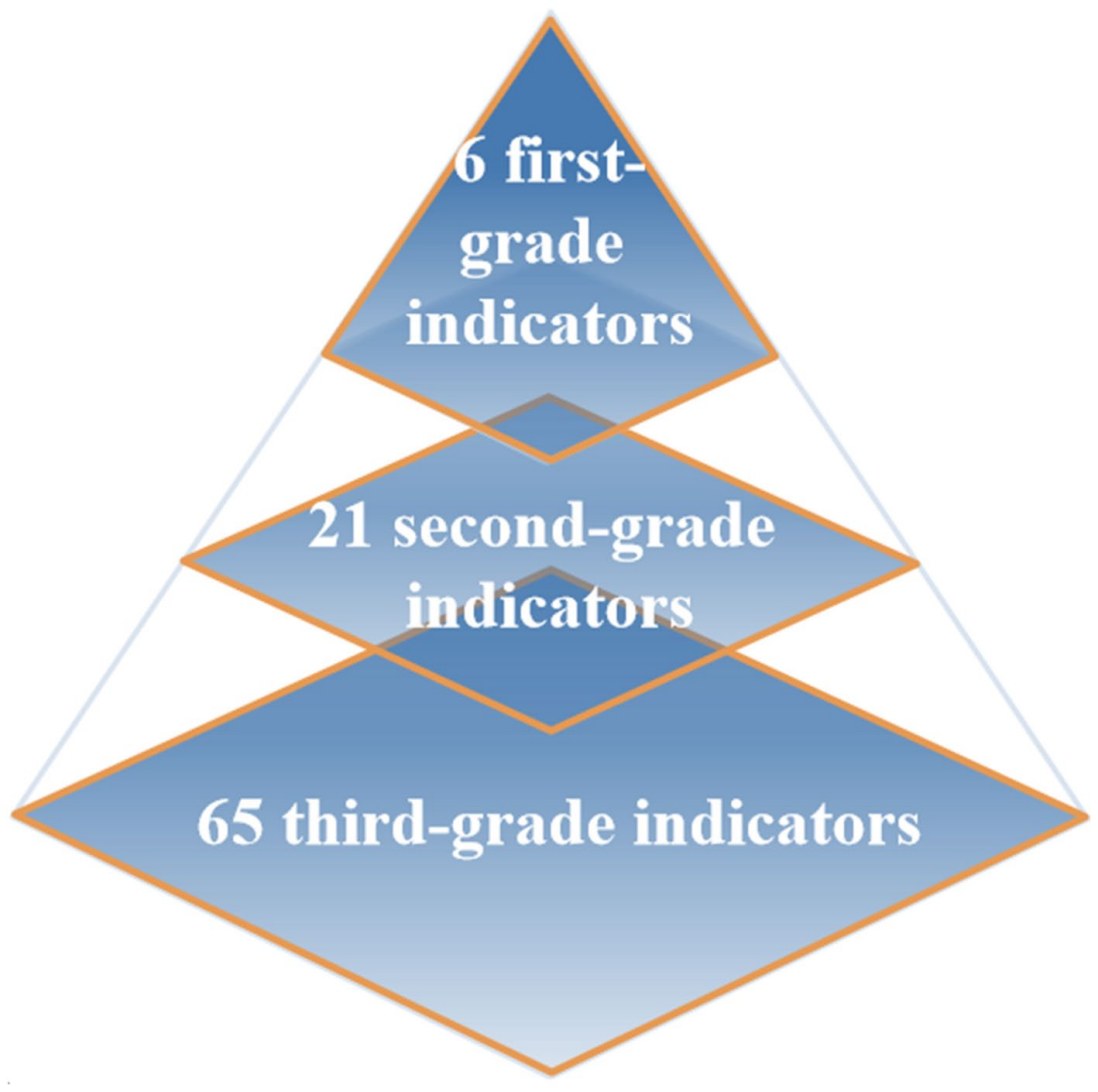
Indicator structure of LDI.

The indicator scheme for LDI comprised of 6 first-grade indicators, 21 s-grade indicators, and 65 third-grade indicators (Figure 1). DEA-CP was used to determine the weights assigned. Table 1 showed the indicator and weight scheme of LDI.

**Table 1:** LDI indicator system.

Grade 1	Weight	Grade 2	Grade 3	Weight	Year of data
Health level	0.327724212722882	Population situation	The proportion of population aged 0–14	0.415935301664094	2020
			The proportion of population aged 15–64	0.051971525168703	2020
			The proportion of population aged 65 and above	0.000002334574056	2020
			Mortality	0.000000035682448	2021
			Natural population growth rate	0.000000103274644	2021
		Health status	Per capita life expectancy	0.415935331456722	2020
			System management rate for children under 3 years old	0.132744338799394	2021
			Maternal system management rate	0.131932745180370	2021
			Prenatal examination rate	0.239183524037821	2021
Medical capabilities	0.149999991707872	Institution	Number of comprehensive hospitals per 10,000 people	0.253278080123479	2021
			Number of Traditional Chinese Medicine Hospitals per 10,000 People	0.000000008952145	2021
			Number of specialized hospitals per 10,000 people	0.000000007270905	2021
			Number of tertiary hospitals per 10,000 people	0.180000000839937	2021
			Number of secondary hospitals per 10,000 people	0.093539539645429	2021
			Number of primary hospitals per 10,000 people	0.176720074433797	2021
			Number of primary healthcare institutions per 10,000 people	0.203491246713378	2021
			Number of maternal and child health clinics/stations per 10,000 people	0.000000007091724	2021
			Number of emergency centers per 10,000 people	0.082803337728690	2021
			Number of sanatoriums per 10,000 people	0.116860529848141	2021
			Number of beds per thousand people in medical institutions	0.214355034991231	2021
		Personnel	Number of pharmacists per 10,000 people	0.000000002095553	2021
			Number of technicians per 10,000 people	0.000000002455737	2021
			Number of rural doctors and healthcare workers per 10,000 people	0.163487032251264	2021
			Number of health technicians per thousand people	0.000000002428709	2021
			Number of practicing (assistant) physicians per thousand people	0.123295108955931	2021
			Registered nurses per thousand people	0.000000040852497	2021
		Service	Annual average number of visits by residents	0.068634495829494	2021
			Annual hospitalization rate of residents	0.000000025487552	2021
			Hospital bed utilization rate	0.000000002739868	2021
			Average length of hospital stay	0.123295106779282	2021
Disease prevention and control	0.149999991530173	Prevention and control guarantee	Incidence rate of Class A and B notifiable infectious diseases	0.477229968505150	2021
			Class A and Class B statutory reported infectious disease mortality rate	0.390205796288109	2021
			Number of CDC personnel per 10,000 people	0.352819480989386	2021
			Number of disease prevention and control centers per 10,000 people	0.000000576018228	2021
			Per capita annual health check ups	0.178032184107061	2021
		Health education	Health education per 10,000 people (institution/station)	0.052833723115696	2021
			Number of public health education activities per 10,000 people	0.062815763238227	2021
Ecological environment	0.094999992262849	Water quality	Urban water supply penetration rate	0.000000052489771	2021
			The water quality compliance rate of centralized drinking water source areas in prefecture level and above cities	0.150000032826464	2021
		Air quality	Days of good air quality in major cities	0.000000031315435	2021
		Greening situation	Urban per capita park green space area	0.179999081034425	2021
			Forest coverage rate	0.180000008339637	2021
		Waste treatment	Urban sewage treatment rate	0.141410953036154	2021
			Total emissions of major pollutants per 10,000 people	0.083694479590853	2021
			Comprehensive utilization rate of general industrial solid waste	0.000000011634061	2021
			Harmless treatment rate of urban household waste	0.145312020303276	2021
		Natural calamities	The proportion of natural disaster deaths to the total population	0.045511448473591	2021
			The proportion of natural disaster victims to the total population	0.000000004075498	2021
		Other related matters	Number of sudden environmental incidents	0.073014973679382	2021
			Altitude	0.130000040818452	2021
Health expenditure	0.100000024509426	Economic development	Per capita regional gross domestic product	0.000000012571049	2021
			Per capita disposable income of residents	0.000000007146529	2021
		Medical expenses	The proportion of total health expenses to GDP	0.149999977641731	2020
			Per capita total health expenses	0.400000003467838	2020
			The proportion of personal health expenditure to total health expenses	0.000000005104950	2020
		Medical insurance	Basic medical insurance fund income per 10,000 people	0.000000006584571	2021
		Birth insurance	The proportion of people enjoying maternity insurance benefits	0.000000015919818	2020
		Medical assistance	The proportion of beneficiaries participating in basic medical insurance	0.175002579995614	2020
			The amount of funding per 10,000 people to participate in basic medical insurance	0.000000135310661	2020
			The proportion of outpatient and inpatient medical assistance recipients to the total population	0.044709025952675	2020
			Outpatient and inpatient medical assistance funds per 10,000 people	0.310358147671988	2020
Health industry	0.180000007011142	Health institution expenditure	Total expenditure of medical and health institutions per 10,000 people	0.268397528567811	2021
		Health product market	Total sales of drugs	0.457097995845349	2021
			Total sales of medical equipment	0.444561956797429	2021
		Pension industry	Number of nursing beds per 10,000 people	0.268397519350026	2021

### 3.2 LDI pilot analysis

We calculated the LDI score for the provinces included (Table 2).

**Table 2:** LDI score of provinces in China.

Zone	Province	LDI	Health level	Medical capabilities	Disease prevention and control	Ecological environment	Health expenditure	Health industry
High security zone	Beijing	100.00	100.00	100.00	100.00	97.13	100.00	100.00
	Shanghai	91.39	94.03	96.41	100.00	76.58	78.45	88.82
	Guangdong	87.76	100.00	72.95	73.89	95.29	59.15	99.99
	Zhejiang	84.95	94.66	100.00	90.27	94.01	55.40	60.63
	Jiangsu	84.57	92.71	93.95	97.07	83.13	49.27	70.61
Medium to high security zone	Shandong	83.12	96.75	84.39	88.66	90.48	57.02	61.98
	Tianjin	82.75	98.02	100.00	95.54	76.41	63.35	42.79
	Hubei	77.63	83.18	85.28	84.81	85.05	55.55	62.35
	Henan	77.05	89.14	75.08	82.95	81.84	52.31	61.81
	Jilin	75.89	79.65	99.80	97.82	83.05	62.14	33.53
	Shaanxi	75.69	92.96	90.96	96.16	82.44	58.23	19.45
	Ningxia	75.57	100.00	87.46	100.00	68.43	50.32	17.46
Medium security zone	Jiangxi	74.36	99.10	83.01	70.30	99.93	45.78	26.76
	Heilongjiang	74.29	75.36	99.75	96.00	87.91	63.10	30.94
	Fujian	74.26	97.67	77.12	81.88	100.00	46.21	23.79
	Hebei	72.97	91.51	65.97	89.58	88.06	53.89	32.71
	Anhui	72.62	86.95	87.73	65.40	87.00	39.73	49.55
	Hunan	72.12	94.20	80.48	54.82	91.79	51.38	39.41
	Hainan	71.74	96.68	94.67	62.41	96.87	61.21	6.53
	Liaoning	71.14	75.86	100.00	82.62	86.57	52.45	30.09
	Xinjiang	70.81	99.84	92.27	67.83	66.49	53.23	13.54
	Sichuan	70.81	86.62	87.43	60.46	78.91	54.05	40.76
	Inner Mongolia	70.08	82.61	99.16	88.35	75.82	49.70	15.03
Low to medium security zone	Shanxi	69.01	85.54	78.66	83.60	66.49	57.24	25.56
	Chongqing	68.72	85.70	82.08	56.16	81.03	44.35	43.14
	Guizhou	67.51	91.85	93.16	55.25	88.22	39.41	15.71
	Yunnan	67.31	79.66	100.00	67.02	77.05	41.90	25.77
	Gansu	66.98	83.70	97.19	94.95	68.17	32.58	5.54
Low security zone	Guangxi	64.20	93.60	79.28	34.80	91.04	46.92	17.06
	Qinghai	60.84	80.87	100.00	65.19	50.64	38.20	5.17
	Tibet	54.75	46.55	75.21	100.00	54.01	62.84	9.97

As shown in Table 2, the highest value of the LDI was 100.00 and the lowest value was 54.75. China mainland was divided into 5 life-safety guarantee zones according to the score, including high security zone, medium to high security zone, medium security zone, low to medium security zone, and low security zone. Provinces with scores greater than or equal to 84 were defined as high security zones, including Beijing, Shanghai, Guangdong, Zhejiang, and Jiangsu, and provinces with scores between 75 and 84 were defined as the medium to high security zone, including Shandong, Tianjin, Hubei, Henan, Jilin, Shaanxi, Ningxia, and provinces with scores between 70 and 75 were defined as the medium security zone, including Jiangxi, Heilongjiang, Fujian, Hebei, Anhui, Hunan, Hainan, Liaoning, Xinjiang, Sichuan, Inner Mongolia, and provinces with scores between 65 and 70 were defined as low to medium security zone, including Shanxi, Chongqing, Guizhou, Yunnan and Gansu, and provinces with scores less than 65 were defined as low security zone, including Guangxi, Qinghai and Tibet.

**Figure 2:**
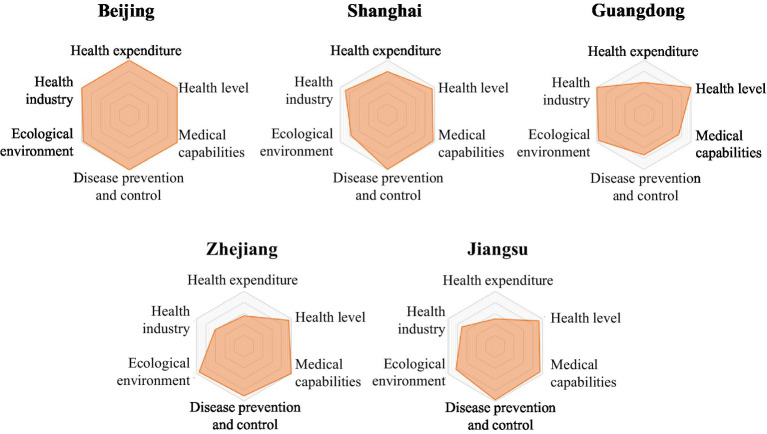
Radar map of LDI in high security zone.

**Figure 3:**
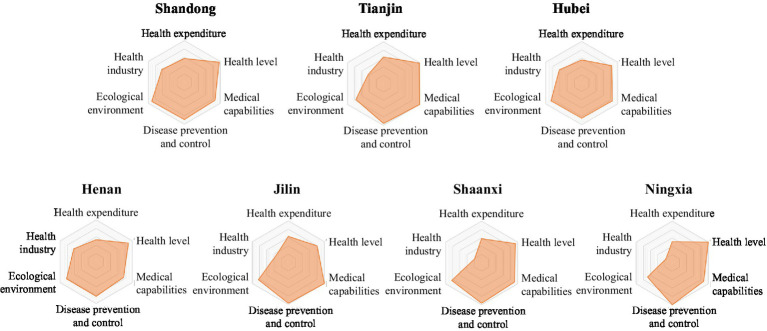
Radar map of LDI in medium to high security zone.

**Figure 4:**
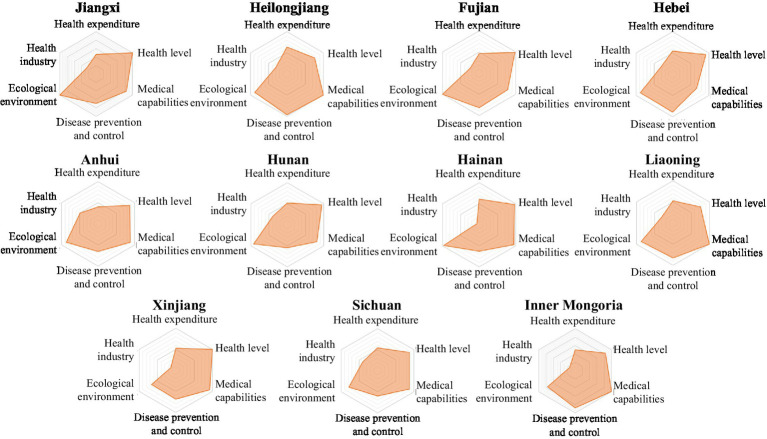
Radar map of LDI in medium security zone.

**Figure 5:**
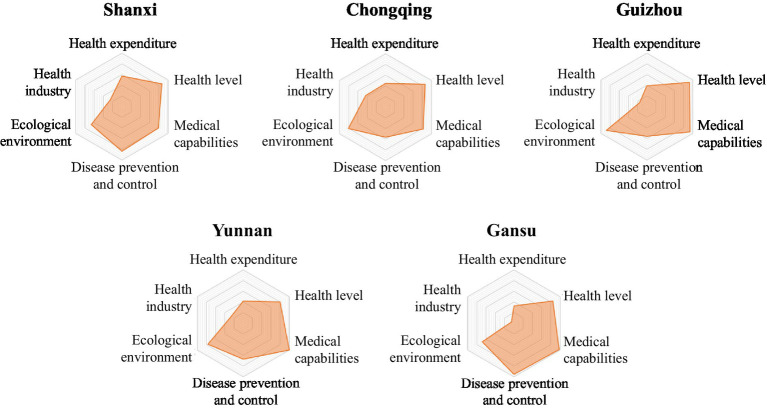
Radar map of LDI in low to medium security zone.

**Figure 6:**
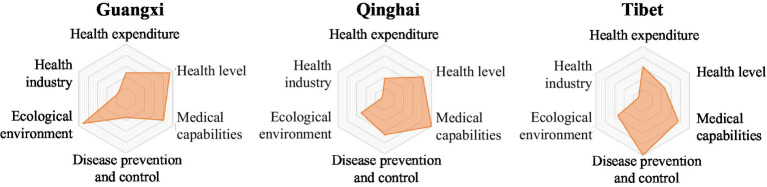
Radar map of LDI in low security zone.

The LDI indicator system comprises six first-level indicators. Based on the scores of the six first-level indicators for each province in Table 2, a six-element radar chart has been created for each province. This chart provides a more intuitive representation of the performance of each region’s six first-level indicators, highlighting their strengths and weaknesses (Figures 2–6).

#### 3.2.1 High security zone

The common characteristics of provinces in the high security zone were high health level, strong medical security capabilities, perfect disease prevention and control system, good ecological environment, adequate medical spending, and developed health industry. The six first-level indicators were balanced and efficient, and the shortcomings in ensuring life-safety were not obvious, or the shortcomings were easy to make up (Figure 2).

1  The LDI of Beijing was 100.00, ranking first. The indices of medical expenditure and health industry both were 100.00, ranking first. The indices of health level, medical capacity, disease prevention and control, and ecological environment were 100.00, 100.00, 100.00 and 97.13 respectively, ranking second, third, fourth and third.
2  The LDI of Shanghai was 91.39, ranking second. The medical expenditure index was 78.45, ranking second; the health industry index was 88.82, ranking third; the disease prevention and control index was 100.00, ranking third; the medical capacity index was 96.41, ranking 11th; the health level index was 94.03, ranking 12th; and the ecological environment index was 76.58, ranking 23rd.
3  The LDI of Guangdong province was 87.76, ranking second. The health industry index was 99.99, ranking second; the health level index was 100.00, ranking third; the ecological environment index was 95.29, ranking 5th, the medical expenditure index was 59.15, ranking 8th; the disease prevention and control index was 73.89, ranking 20th; the medical capacity index was 72.95, ranking second-to-last.
4  The LDI of Zhejiang province was 84.95, ranking fourth. The medical capacity index was 100.00, ranking 4th; the ecological environment index was 94.01, ranking 6th; the health industry index was 60.63, ranking 8th; the health level index was 94.66, ranking 10th; the disease prevention and control index was 90.27, ranking 11th; the medical expenditure index was 55.40, ranking 13th.
5  The LDI of Jiangsu province was 84.57, ranking fifth. The health industry index was 70.61, ranking 6th; the disease prevention and control index was 97.07, ranking 6th; the medical capacity index was 93.95, ranking 13th; the health level index was 92.71, ranking 15th; the ecological environment index was 83.13, ranking 16th; the medical expenditure index was 49.27, ranking 22nd.

#### 3.2.2 Medium to high security zone

The common characteristics of provinces in the medium to high security zone were high health level, strong medical security ability, perfect disease prevention and control system construction, and relatively balanced ecological environment construction. However, there was an obvious economic gap with the high security zone, resulting in the relative shortage of resource allocation of the two elements of medical expenditure and health industry. Therefore, this security zone should increase medical investment and strengthen the development of the health industry (Figure 3).

6  The LDI of Shandong province was 83.12, ranking 6th. The health industry index was 61.98, ranking 6th; the health level index was 96.75, ranking 8th; the ecological environment index was 90.48, ranking 9th; the medical expenditure index was 57.02, ranking 11th; the disease prevention and control index was 88.66, ranking 13th; the medical capacity index was 84.39, ranking 21st.
7  The LDI of Tianjin province was 82.75, ranking seventh. The medical expenditure index was 63.35, ranking 3rd; the medical capacity index was 100.00, ranking 5th; the health level index was 98.02, ranking 6th; the disease prevention and control index was 95.54, ranking 9th; the health industry index was 42.79, ranking 11th; the ecological environment index was 76.41, ranking 24th.
8  The LDI of Hubei province was 77.63, ranking eighth. The health industry index was 62.35, ranking 5th; the medical expenditure index was 55.55, ranking 12th; the disease prevention and control index was 84.81, ranking 15th; the ecological environment index was 85.05, ranking 15th; the medical capacity index was 85.28, ranking 20th; and the health level index was 83.18, ranking 24th.
9  The LDI of Henan province was 77.05, ranking ninth. The health industry index was 61.81, ranking 7th; the medical expenditure index and health level index were 52.31 and 89.14 respectively, both ranking 18th; the disease prevention and control index was 82.95, ranking 17th; the ecological environment index was 81.84, ranking 19th; the medical capacity index was 75.08, ranking third to last.
10  The LDI of Jilin Province was 75.89, ranking 10th. The disease prevention and control index was 97.82, ranking 5th; the medical expenditure index was 62.14, ranking 6th; the medical capacity index was 99.80, ranking 7th; the health industry index was 33.53, ranking 14th; the ecological environment index was 83.05, ranking 17th; the health level index was 79.65, ranking fourth.
11  The LDI of Shaanxi province was 75.69, ranking 11th. The disease prevention and control index was 96.16, ranking 7th; the medical expenditure index was 58.23, ranking 9th; the health level index was 92.96, ranking 14th; the medical capacity index was 90.96, ranking 16th; the ecological environment index was 82.44, ranking 18th; the health industry index was 19.45, ranking 22nd.
12  The LDI of Ningxia Hui Autonomous Region was 75.57, ranking 12th. The health level index was 100.00, ranking first; the disease prevention and control index was 100.00, ranking first; the medical capacity index was 87.46, ranking 18th; the medical expenditure index was 50.32, ranking 20th; the health industry index was 17.46, ranking 23rd; the ecological environment index was 68.43, ranking 26th.

#### 3.2.3 Medium security zone

The common characteristics of provinces in the medium security zone were basic balance and medium efficiency between health level, medical ability, disease prevention and control, ecological environment, but the medical ability of Hebei province and the capacity of disease prevention and control of Anhui, Hunan, Hainan, Xinjiang, Sichuan provinces were not strong. The inadequate medical spending and health industry of this security zone were more obvious. Some of the disadvantages in this zone may be easily ameliorated, and some of them require long-term accumulation to be ameliorated (Figure 4).

13  The LDI of Jiangxi province was 74.36, ranking 13th. The ecological environment index was 99.93, ranking 2nd; the health level index was 99.10, ranking 5th; the health industry index was 26.76, ranking 18th; the disease prevention and control index was 70.30, ranking 21st; the medical capacity index was 83.01, ranking 22nd; the medical expenditure index was 45.78, ranking 25th.
14  The LDI of Heilongjiang province was 74.29, ranking 14th. The medical expenditure index was 63.10, ranking fourth; the medical capacity index was 99.75, ranking 8th; the disease prevention and control index was 96.00, ranking 8th; the ecological environment index was 87.91, ranking 12th; the health industry index was 30.94, ranking 16th; and the health level index was 75.36, ranking 30th.
15  The LDI of Fujian province was 74.26, ranking 15th. The ecological environment index was 100.00, ranking first; the health level index was 97.67, ranking 7th; the disease prevention and control index was 81.88, ranking 19th; the health industry index was 23.79, ranking 21st; the medical expenditure index was 46.21, ranking 24th; the medical capacity index was 77.12, ranking 27th.
16  The LDI of Hebei province was 72.97, ranking 16th. The ecological environment index was 88.06, ranking 11th; the disease prevention and control index was 89.58, ranking 12th; the medical expenditure index and the health industry index were 53.89 and 32.71 respectively, both ranking 15th; the health level index was 91.51, ranking 17th; the medical capacity index was 65.97, ranking 31st.
17  The LDI of Anhui province was 72.62, ranking 17th. The health industry index was 49.55, ranking 9th; the ecological environment index was 87.00, ranking 13th; the medical capacity index was 87.73, ranking 17th; the health index was 86.95, ranking 19th; the disease prevention and control index was 65.40, ranking 24th; the medical expenditure index was 39.73, ranking 28th.
18  The LDI of Hunan province was 72.12, ranking 18th. The ecological environment index was 91.79, ranking 7th; the health level index was 94.20, ranking 11th; the health industry index was 39.41, ranking 13th; the medical expenditure index was 51.38, ranking 19th; the medical capacity index was 80.48, ranking 24th; the disease prevention and control index was 54.82, ranking 30th.
19  The LDI of Hainan province was 71.74, ranking 19th. The ecological environment index was 96.87, ranking 4th; the medical expenditure index was 61.21, ranking 7th; the health level index was 96.68, ranking 9th; the medical capacity index was 94.67, ranking 12th; the disease prevention and control index was 62.41, ranking 26th; and the health industry index was 6.53, ranking 29th.
20  The LDI of Liaoning province was 71.14, ranking 20th. The medical capacity index was 100.00, ranking first; the ecological environment index was 86.57, ranking 14th; the medical expenditure index and the health industry index were 52.45 and 30.09 respectively, both ranking 17th; the disease prevention and control index was 82.62, ranking 18th; the health level index was 75.86, ranking 29th.
21  The LDI of Xinjiang Uygur Autonomous Region was 70.81, ranking 21st. The health level index was 99.84, ranking 4th; the medical capacity index was 92.27, ranking 15th; the medical expenditure index was 53.23, ranking 16th; the disease prevention and control index was 67.83, ranking 22nd; the health industry index was 13.54, ranking 27th; and the ecological environment index was 66.49, ranking 29th.
22  The LDI of Sichuan province was 70.81, ranking 22nd. The health industry index was 40.76, ranking 12th; the medical expenditure index was 54.05, ranking 14th; the medical capacity index was 87.43, ranking 19th; the health level index was 86.62, ranking 20th; the ecological environment index was 78.91, ranking 21st; the disease prevention and control index was 60.46, ranking 27th.
23  The LDI of Inner Mongolia was 70.08, ranking 23rd. The medical capacity index was 99.16, ranking 9th; the disease prevention and control index was 88.35, ranking 14th; the medical expenditure index was 49.70, ranking 21st; the health level index and the ecological environment index were 82.61 and 75.82 respectively, both ranking 25th; the health industry index was 15.03, ranking 26th.

#### 3.2.4 Low to medium security zone

The common characteristics of provinces in the low to medium security zone were weak economic development, obvious shortcomings in medical expenditure and health industry, and obviously weak disease prevention and control capacity of Chongqing, Guizhou and Yunnan provinces, and the ecological environment index of Shanxi and Gansu were obviously small due to their geological reasons (Figure 5). Therefore, the five provinces should take economic development as the center, and strengthen medical expenditure and health industry development, but also pay attention to disease prevention and control, as well as ecological environment construction.

24  The LDI of Shanxi Province was 69.01, ranking 24th. The medical expenditure index was 57.24, ranking 10th; the disease prevention and control index was 83.60, ranking 16th; the health industry index was 25.56, ranking 20th; the health level index was 85.54, ranking 22nd; the medical capacity index was 78.66, ranking 26th; the ecological environment index was 66.49, ranking 28th.
25  The LDI of Chongqing was 68.72, ranking 25th. The health industry index was 43.14, ranking 10th; the ecological environment index was 81.03, ranking 20th; the health level index was 85.70, ranking 21st; the medical capacity index was 82.08, ranking 23rd; the medical expenditure index was 44.35, ranking 26th; the disease prevention and control index was 56.16, ranking 28th.
26  The LDI of Guizhou province was 67.51, ranking 26th. The ecological environment index was 88.22, ranking 10th; the medical capacity index was 93.16, ranking 14th; the health level index was 91.85, ranking 16th; the health industry index was 15.71, ranking 25th; the medical expenditure index and disease prevention and control index were 39.41 and 55.25 respectively, both ranking 29th.
27  The LDI of Yunnan province was 67.31, ranking 27th. The medical capacity index was 100.00, ranking 6th; the health industry index was 25.77, ranking 19th; the ecological environment index was 77.05, ranking 22nd; the disease prevention and control index was 67.02, ranking 23rd; the health level index and the medical expenditure index were 79.66 and 41.90 respectively, both ranking 27th.
28  The LDI of Gansu province was 66.98, ranking 28th. The medical capacity index was 97.19, ranking 10th; the disease prevention and control index was 94.95, ranking 10th; the health level index was 83.70, ranking 23rd; the ecological environment index was 68.17, ranking 27th; the health industry index was 5.54, ranking 30th; and the medical expenditure index was 32.58, ranking 31st.

#### 3.2.5 Low security zone

The common characteristics of provinces in the low security zone were insufficient medical expenditure, low health level, poor medical capacity, weak disease prevention and control, poor ecological environment and weak health industry (Figure 6). The ability of this zone to protect people’s life-safety was poor, which requires the comprehensive force of multiple elements. However, at the same time, some disadvantages of Qinghai and Tibet were caused by external factors such as geography, environment and altitude that cannot be changed. Therefore, these three provinces should make up for the factors that they can change, and try their best to narrow the gap with other provinces.

29  The LDI of Guangxi Zhuang Autonomous Region was 64.20, ranking 29th. The ecological environment index was 91.04, ranking 8th; the health level index was 93.60, ranking 13th; the medical expenditure index was 46.92, ranking 22nd; the health industry index was 17.06, ranking 24th; the medical capacity index was 79.28, ranking 25th; the disease prevention and control index was 34.80, ranking 31st.
30  The LDI of Qinghai province was 60.84, ranking 30th. The medical capacity index was 100.00, ranking second; the disease prevention and control index was 65.19, ranking 25th; the health level index was 80.87, ranking 26th; the medical expenditure index was 38.20, ranking 30th; the ecological environment index and the health industry index were 50.64 and 5.17 respectively, both ranking 31st.
31  The LDI of Tibet Autonomous Region was 54.75, ranking 31st. The disease prevention and control index was 100.00, ranking second; the medical expenditure index was 62.84, ranking fifth; the medical capacity index and the health industry index were 75.21 and 9.97 respectively, both ranking 28th; the ecological environment index was 54.01, ranking 30th; the health level index was 46.55, ranking 31st.

## 4 Discussion

By reviewing more than 1,200 literatures, we found that there are two types of health index. One type index is to evaluate the health status of major countries around the world and form a global index distribution, such as the Global Health Security Index (GHS) (10), the Human Development Index (HDI) (11), the Global One Health Index (GOHI) (12, 13), the Bloomberg Global Health Index (14), European Health Index (15), International Dietary Health Index (16). Another type index is to evaluate the health status of cities or regions in one country, aiming to understand the health level of the people in one country, such as the “China Health Index” (17), the “England Health Index” (18, 19), and the “Iran Comprehensive Social Health Index” (20). However, these studies have primarily focused on health, rather than life-safety. There is no unified concept of life-safety, and the relationship between life-safety and health is not clear. This study gives the concept of life-safety, and clarifies the relationship between life-safety and health. The indicator system of LDI is divided into three levels, providing clear determinants for the assessment of life-safety status and promoting a common understanding of these determinants.

At present, the methods of assigning weights to indicators mainly include experts grading method, analytic hierarchy process (AHP), TOPSIS method, or equal weight method. AHP requires manual setting of indicator weights. TOPSIS relies on preset weights. Although the equal weight method avoids the subjective error of artificial weighting, it loses the flexibility of the evaluation method. There are certain subjective factors and subjective errors in the processing process of these methods. DEA-CP combines the flexibility of DEA (first stage) with the global optimization of compromise programming (second stage), effectively addressing the challenge of weight allocation in multi-attribute comprehensive evaluation. Meanwhile, the use of the L2 norm ensures a unique solution to the model, avoiding the issue of multiple evaluation units tying for first place in traditional DEA, thereby improving the distinction in rankings. This study is the first to use the DEA-CP model, which can intelligently assign weights to each indicator, avoiding subjective errors and ensuring the objectivity and accuracy of evaluation results.

To ensure the validity of the data, we use authoritative data publicly available by the Chinese government or provinces as the main source of LDI data, which may result in limitations on the inclusion of some indicators. Due to a lack of data (such as data on indicators related to personal lifestyle habits), some sensitive indicators were not included in the analysis.

According to the LDI framework analysis (Table 1), medical capabilities and disease prevention-control demonstrate substantial weighting coefficients, which substantiates their critical role in safeguarding life-safety. It is suggested to increase expenditure on public health services, increase the construction of public infrastructure to ensure the life-safety of people, strengthen public propaganda, enhance people’s awareness of life-safety, and improve people’s lifestyle through reasonable diet, moderate exercise, smoking and drinking cessation, and psychological balance. Strengthening the construction of the public health system is also an essential measure to ensure the life-safety, including enhancing the ability to respond to sudden infectious diseases, and increasing the prevention and control of chronic diseases through improving the comprehensive prevention and control mechanism of chronic diseases, promoting integrated services for prevention, treatment, and rehabilitation, and other means.

## 5 Conclusion

Ensuring life-safety requires the joint collaboration of the central government, provinces, cities, and individuals in economic, medical, environmental, individual, and social aspects. Health level is the core manifestation of life-safety, medical capacity as well as disease prevention and control are important means to ensure life-safety. The ecological environment is an external factor that ensures life-safety. The medical expenditure and health industry determined by the economy are the foundation of life-safety guarantee. Individuals, including factors such as diet, exercise, psychology, and genetics, are important factors in ensuring people’s health. A stable social environment with complete policies and conducive to safeguarding life-safety is crucial.

From LDI, it can be seen that China as a country with a large population and vast territory, there is a significant gap in the ability of each province to ensure the life-safety. The disparity in economic foundations has led to the concentration of medical resources in developed provinces, resulting in an uneven distribution of medical resources among regions. Therefore, it is suggested that the government can attract high-quality medical resources to stay in underdeveloped areas through policy guidance, such as improving the treatment of doctors in underdeveloped areas, optimizing the evaluation criteria for doctor titles, etc. At the same time, by establishing intelligent internet hospitals, we can bridge the gap between hospitals and patients, so that people in underdeveloped areas and grass-roots people can also get high-quality medical services.

LDI is the world’s first research tool that builds an evaluation framework from a holistic perspective of life-safety. LDI can be utilized to quantitatively evaluate the life-safety performance within each country globally, and pinpoint disparities in ensuring people’s life-safety across regions within that country, thereby providing a solid foundation for policy-making in each nation. If LDI is intended to assess the life-safety status and disparities between countries or across the globe, it must take into account the influence of the global economy, policies, and the mutual interactions among nations on the life-safety status, which requires adjusting the indicator system of the index.

In the future, we will establish an indicator system suitable for evaluating the life-safety status among countries, and then employ the DAE-CP method to assess the life-safety status of each country, aiming to enhance global people’s life-safety and facilitate the establishment of a community with a shared future for mankind.

## Data Availability

The original contributions presented in the study are included in the article/supplementary material, further inquiries can be directed to the corresponding author.
