# Correction: Life-safety disparity index: a comprehensive, valid, and reliable tool to assess Chinese people's life-safety performance

**DOI:** 10.3389/fpubh.2025.1649485

**Published:** 2025-07-09

**Authors:** Xiaoyue Ge, De an Wu, Xinming Shi, Junxiang Zhang, Jinghui Jiang, Bicheng Zhang, Hongguang Wang

**Affiliations:** ^1^The Academy of Chinese Health Risks of West China Hospital, Sichuan University, Chengdu, China; ^2^School of Mathematics, University of Electronic Science and Technology of China, Chengdu, China

**Keywords:** life-safety, health, life-safety disparity index, model, China, life-safety guarantee zone

In the published article, there was an error in the Funding statement. It originally stated: “The author(s) declare that no financial support was received for the research and/or publication of this article.” The correct Funding statement appears below.

